# A Four-MicroRNA Panel in Peripheral Blood Identified as an Early Biomarker to Diagnose Acute Myocardial Infarction

**DOI:** 10.3389/fphys.2021.669590

**Published:** 2021-07-07

**Authors:** Liang Chen, Jie Bai, Jun Liu, Huihe Lu, Koulong Zheng

**Affiliations:** Department of Cardiology, The Second Affiliated Hospital of Nantong University, Nantong, China

**Keywords:** AMI, peripheral blood, a four-microRNA panel, novel biomarker, diagnosis

## Abstract

**Objective:** This study aimed to evaluate suitable circulating microRNAs (miRNAs) as diagnostic biomarkers of acute myocardial infarction (AMI).

**Methods:** Patients with AMI were enrolled as study participants. All patients with AMI coming from the Second Affiliated Hospital of Nantong University between October 1, 2017 and May 31, 2019 were screened. At the same time, 80 patients with coronary angiographic stenosis <50% during the same period were selected as the control group. Peripheral blood samples were collected at different time points (0, 6, 12, and 24 h after disease onset) to detect the expression of a previously identified promising four-microRNA panel. The expression levels of miRNAs were tested by real-time polymerase chain reaction (RT-PCR), and the receiver operating characteristic curve (ROC) was used to analyze the diagnostic value of circulating miRNAs.

**Results:** Based on the inclusion and exclusion criteria, 80 patients with AMI and 80 controls were enrolled in this study. The expression of circulating miR-1291, miR-217, miR-455-3p, and miR-566 was significantly downregulated in patients with AMI compared with controls. The area under the ROC curve (AUC) of circulating miR-1291, miR-217, miR-455-3p, and miR-566 were 0.82, 0.79, 0.82, and 0.83, respectively. The AUC of these four miRNAs was 0.87 with 83% sensitivity and 87% specificity. The expression peaks of these four miRNAs occurred earlier than those of cardiac troponin I (cTnI) and creatine kinase-MB (CK-MB). Furthermore, Kyoto Encyclopedia of Genes and Genomes (KEGG) pathway analysis showed that the targets of these four miRNAs were significantly enriched in several signaling pathways associated with AMI progression.

**Conclusion:** Circulating miR-1291, miR-217, miR-455-3p, and miR-566 expression levels were significantly lower in patients with AMI; and combined, this panel of four miRNAs acted as a novel and potential early diagnostic biomarker of AMI.

## Introduction

Cardiovascular disease has become a major threat to human health. As one of the most common and severe cardiovascular diseases, acute myocardial infarction (AMI) has become one of the main causes of sudden death in adults because of its acute onset, rapid progress, and high mortality (Reynolds et al., 2017). It is estimated that 16 million people will suffer from AMI in 2020 and 23 million in 2030 (Moran et al., 2010). Therefore, rapid and accurate diagnosis of AMI can improve the course, therapeutic effect and survival rate. At present, biological spectrum markers, such as creatine kinase-MB (CK-MB) and cardiac troponin I (cTnI), have been widely used in the clinical diagnosis of AMI. Due to limitations in specificity, they are not sufficiently efficient to perform an early diagnosis of AMI (de Winter et al., 1995; de Lemos et al., 2010). Therefore, seeking novel, sensitive, and specific biomarkers for the early diagnosis of AMI is needed urgently.

MicroRNAs (miRNAs), a class of small non-coding RNA molecules, are 19–25 nucleotides in length and regulate gene expression at the post-transcriptional level through direct binding to the 3′-UTR of target mRNAs, leading to the inhibition of mRNA translation or mRNA degradation (Filipowicz et al., 2008). At present, the number of human miRNAs exceeds 1,900, and the number of target genes is predicted to be in the range of 1,000 s. Some estimates indicate that miRNAs target more than 30% of the human genome (Escuin et al., 2020). MiRNAs can be produced by all cell types and come from a variety of cell sources, such as endothelial cells, monocytes and macrophages, vascular smooth cells and platelets, and possibly secreted in the blood encapsulated in microparticles (exosomes, microvesicles, and apoptotic bodies), or combined with proteins or high-density lipoproteins (HDL) (Navickas et al., 2016). MiRNAs are stable in peripheral blood and can be identified as biomarkers for disease diagnosis (Navickas et al., 2016). Recently, the role of miRNAs in the diagnosis of AMI has attracted increasing attention (Cheng et al., 2010; Li et al., 2012). Circulating miRNAs, such as miR-499, miR-328, miR-134, miR-208a, miR-1, and miR-126, were dysregulated in AMI and served as potential novel indicators for AMI (Long et al., 2012; He et al., 2014; Xiao et al., 2014). This study aimed to comprehensively select miRNAs, which are significantly dysregulated in peripheral blood during AMI and to identify a panel of miRNAs that could be used as an early diagnostic biomarker of AMI.

## Methods

### Ethics Statement

This study was carried out at the Second Affiliated Hospital of Nantong University in Jiangsu, China. Peripheral blood samples were collected at different time points (0, 6, 12, and 24 h after disease onset) from patients attending to the Second Affiliated Hospital of Nantong University. The study was conducted in accordance with the principles of the Declaration of Helsinki. The study design was approved by the ethical committee of the Second Affiliated Hospital of Nantong University (2019KN109).

### Patients

A total of 80 patients with AMI and 80 patients with coronary angiographic stenosis of <50%, who formed the control group, came from the Department of Cardiology, the Second Affiliated Hospital of Nantong University between October 1, 2017 and May 31, 2019. Age, sex, course of the disease, and other general information were recorded for all participants. The clinical characteristics of patients are shown in Table 1.

**Table 1:** Clinical characteristics of patients with AMI and controls.

**Characteristics**	**Patients with AMI**		**Controls**	***P*-value**
	**STEMI (*n* = 45)**	**NSTEMI (*n* = 35)**	**(*n* = 80)**	
Age (year)	56.347 ± 11.37	58.03 ± 12.18	57.49 ± 12.01	0.618
Male/Female (*n*/*n*)	34/11	28/7	59/21	0.713
Smoking (y/n)	7/38	5/30	9/71	0.640
Hyperlipidemia (y/n)	9/36	4/31	10/70	0.653
Hypertension (y/n)	26/19	11/24	34/46	0.750
Diabetes (y/n)	9/36	7/28	13/67	0.682
Total cholesterol (mmol/L)	3.87 ± 1.12	4.03 ± 1.10	3.94 ± 1.06	0.874
Total triglyceride	1.36 ± 1.09	1.41 ± 0.91	1.32 ± 0.48	0.389
HDL (mmol/L)	1.07 ± 0.31	1.14 ± 0.29	1.06 ± 0.27	0.073
LDL (mmol/L)	2.48 ± 1.07	2.39 ± 0.91	2.54 ± 0.97	0.512
SBP (mm Hg)	123.47 ± 20.48	124.32 ± 20.19	125.16 ± 21.07	0.639
DBP (mm Hg)	77.54 ± 12.18	74.48 ± 10.61	76.43 ± 11.77	0.147
CK-MB (U/L)	59.63 ± 87.55	49.62 ± 60.23	15.08 ± 7.92	**<0.001**
cTnI (ng/mL)	1.02 ± 1.31	1.21 ± 1.29	0.04 ± 0.14	**<0.001**

*AMI, acute myocardial infarction; CK-MB, creatine kinase-MB; cTnI, cardiac troponin I; DBP, diastolic blood pressure; HDL, high-density lipoprotein; LDL, low-density lipoprotein; NSTEMI, non-ST-segment elevation myocardial infarction; SBP, systolic blood pressure; STEMI, ST-segment-elevation myocardial infarction. P-value, comparison between patients with AMI and healthy controls. Bold values indicates statistically significant difference P < 0.05*.

Patients with AMI (ST-segment-elevation myocardial infarction, STEMI and non-ST-segment elevation myocardial infarction, NSTEMI) were identified based on the diagnosis of AMI in accordance with the fourth edition of the global unified definition of MI released at the annual meeting of the European Society of Cardiology (Benjamin et al., 2019). The inclusion criteria were as follows: (1) post-sternal pain with a time longer than 30 min; (2) typical signs of myocardial ischemia on ECG; (3) upregulation of cTnI and CK-MB; (4) no relief from continuous chest pain after taking nitrate drugs; (5) admission of patients within 12 h of chest pain onset; and (6) patients with the primary onset of AMI. The exclusion criteria were as follows: (1) previous MI; (2) previous use of thrombolytic agents for MI; (3) cardiomyopathy cardiogenic shock; (4) previous stroke in the last 6 months; and (5) known bleeding diathesis. Patients were excluded if the chest pain was considered to be caused by trauma, drugs, and medical intervention.

### Sample Collection

Random blood specimens were collected from patients with AMI at different time points after the onset of AMI (0, 6, 12, and 24 h). Plasma was collected from venous blood in blood collection bottles containing ethylenediaminetetraacetic acid (EDTA) after being centrifuged at 4,000 rpm for 10 min and, subsequently, stored at −80°C until further analysis. Total RNA was isolated from plasma using TRIzol LS reagent (assay ID:10296028, Invitrogen, CA, USA) following the protocol of the manufacturer. Cel-miR-39 (assay ID:000200, GenePharma, Shanghai, China) was added to each sample at a final concentration of 10^−4^ pmol/μl acting as the external reference.

### RNA Extraction

The plasma RNA was extracted by using Trizol LS reagent (Invitrogen, USA) according to the protocols of the manufacturer. Briefly, 250 μl plasma samples were transferred into a 1.5 ml centrifuge tube and mixed with 1 ml Trizol LS reagent at room temperature for 5 min. Then, 200 μl chloroform was added to a centrifuge tube and shaken for 15 s and was allowed to stand for 2 min. Next, the samples were centrifuged at 12,000 × *g* for 5 min at 4°C, and the supernatant was collected in another centrifuge tube. The supernatant was mixed gently with 0.5 ml isopropanol and was allowed to stand at room temperature for 10 min. Then, the samples were again centrifuged at 12,000 × *g* for 10 min at 4°C and the supernatant was discarded. The sediment was cleaned gently with 75% ethanol, and the supernatant was discarded. Finally, the sediment was dried, and an appropriate amount of RNase-free water was added.

### Quantitative Reverse Transcription-Polymerase Chain Reaction (qRT-PCR)

Reverse transcription and quantitative reverse transcription-polymerase chain reaction for miR-1291, miR-217, miR-455-3p, miR-566, and external reference miR-39 were performed using a Hairpin-itTM miRNA RT-PCR Quantitation Kit (assay ID: E01007, GenePharma, Shanghai, China) following the protocols of the manufacturer. First, reverse transcription was conducted by using 2 μg total RNA to get cDNA. The reverse transcription program was set at 25°C for 30 min, followed by 42°C for 30 min, and 85°C for 5 min. Then, qRT-PCR was carried out. The qRT-PCR reactions were initiated with denaturation at 95°C for 3 min, followed by 40 cycles of 95°C for 15 s, and 62°C for 34 s. The primers of each miRNA were as follows: forward and reverse primers of miR-1291 were 5′-CCTGACTGAAGACCAGC-3′ and 5′-GAACATGTCTGCGTATCTC-3′; forward and reverse primers of miR-217 were 5′-TACTGCATCAGGAACTGA-3′ and 5′-GAACATGTCTGCGTATCTC-3′; forward and reverse primers of miR-455-3p were 5′-GTGCCTTTGGACTACATC-3′ and 5′-GAACATGTCTGCGTATCTC-3′; forward and reverse primers of miR-566 were 5′- GGGCGCCUGUGAUCCCAAC-3′ and 5′-UGGGAUCACAGGCGCCCUU-3′; and forward and reverse primers of cel-miR-39 were 5′-CCAGCTCACCGGG-TGTAAA-3′ and 5′-AGCAGGGTCCGAGGTATTC-3′. The relative expression levels of miR-1291, miR-217, miR-455-3p, and miR-566 were calculated by using the 2^−ΔΔCt^ method. ΔCt = Ct sample – Ct cel-miR-39 and ΔΔCt = ΔCt _AMI_ – ΔCt control.

### Biochemical Assays

The expression levels of circulating cTnI were measured by using a Roche high-sensitivity assay performed on a Cobas C8000 system (Roche Diagnostics, Germany). CK-MB was also measured on a Cobas C8000 instrument by using a Roche International Federation of Clinical Chemistry (IFCC)-recommended method.

### Bioinformatics Analysis

The microarray assay used a public Gene Expression Omnibus (GEO) dataset (GSE31568) (https://www.ncbi.nlm.nih.gov/geo/). GSE31568 contains 70 healthy controls and 20 patients with AMI, whose platform is GPL9040 (febit Homo Sapiens miRBase 13.0). The differentially expressed miRNAs in GSE31568 were analyzed by using the R package “Limma.” The targets of miRNAs were searched by integrated analysis of the TargetScan 7.2 database (http://www.targetscan.org/vert_72/) and miRDB database (http://mirdb.org). Kyoto Encyclopedia of Genes and Genomes (KEGG) pathway analysis was conducted by using DAVID Bioinformatics Resources 6.8 (https://david.ncifcrf.gov).

### Statistical Analysis

The clinical characteristics of patients with AMI and controls were compared using the chi-square test. The differential expression levels of miRNAs among groups were determined using ANOVA or the Student's unpaired-sample test. Receiver operating characteristic (ROC) curve and area under the ROC curve (AUC) were established for discriminating patients with AMI from controls. A cutoff value of the expression levels of miR-1291, miR-217, miR-455-3p, and miR-566 was determined using the Youden index from ROC curves. A *P* < 0.05 indicated a statistically significant difference. Statistical analysis was performed using GraphPad 5.0 (GraphPad Software, CA, USA).

## Results

### Integrated Analysis of Microarray Assay Identified miRNAs Holding Potential as Diagnostic Biomarkers of AMI

**Figure 1:**
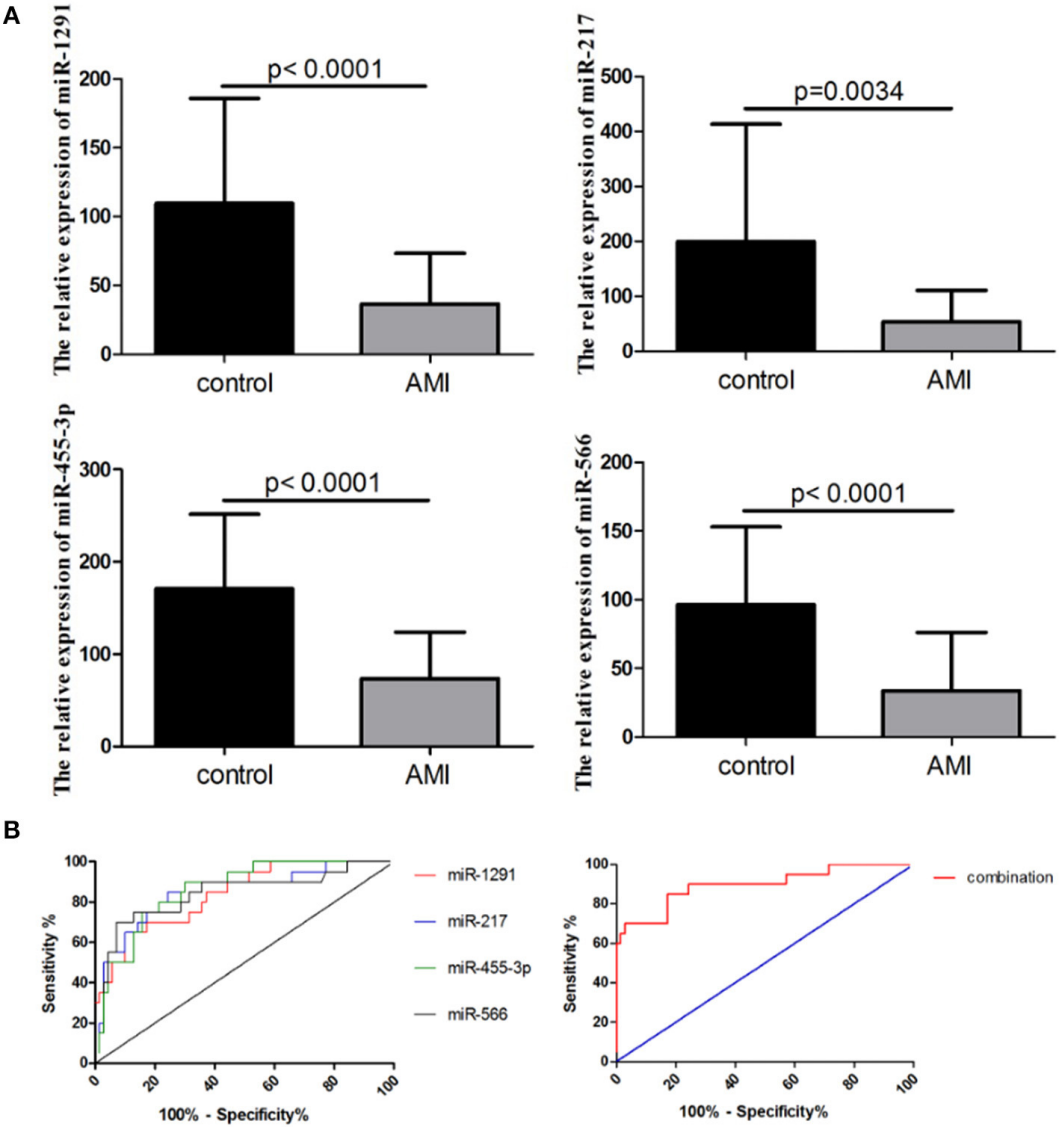
Expression of a four-microRNA (miRNA) panel in GSE31568. **(A)** Relative expression of miR-1291, miR-217, miR-455-3p, and miR-566 in the plasma of patients with AMI in GSE31568. **(B)** Receiver operating characteristic (ROC) curves of miR-1291, miR-217, miR-455-3p, and miR-566, and the combination of four miRNAs in GSE31568.

The integrated analysis of the GSE31568 dataset revealed that miR-1291, miR-1303, miR-217, mir-23b^*^, miR-380^*^, miR-455-3p, miR-492, miR-566, miR-624, miR-636, and miR-920 were significantly dysregulated in the plasma of patients with AMI (Table 2). ROC curve was used to investigate the diagnostic utility of these miRNAs, revealing that the AUCs of miR-1291, miR-217, miR-455-3p, and miR-566 were higher than those of other miRNAs (Table 2). The relative expression levels of miR-1291, miR-217, miR-455-3p, and miR-566 were significantly downregulated in the plasma of patients with AMI compared with controls in GSE31568 (Figure 1A). The AUC of miR-1291, miR-217, miR-455-3p, and miR-566 was 0.84 (*P* < 0.001), 0.825 (*P* < 0.001), 0.86 (*P* < 0.001), and 0.84 (*P* < 0.001), respectively. Interestingly, the combination of these four miRNAs resulted in a higher AUC value of 0.90 (*P* < 0.001) with 85% sensitivity and 82.86% specificity (Figure 1B). These data suggested that the combination of miR-1291, miR-217, miR-455-3p, and miR-566 could serve as a novel potential diagnostic biomarker of AMI.

**Table 2:** Relative expression and diagnostic value of significantly dysregulated miRNAs in the plasma of patients with AMI in GSE31568.

**miRNAs**	**logFC**	***P*-value**	**AUC**	**95% CI**	***P*-value**
miR-1291	−1.39	0.0384	**0.84**	0.74–0.93	**<0.0001**
miR-1303	1.88	0.016	0.75	0.60–0.89	**0.00085**
miR-217	−1.38	0.0104	**0.83**	0.75–0.95	**<0.0001**
miR-23b*	1.64	0.048	0.79	0.66–0.92	**<0.0001**
miR-380*	−3.46	<0.0001	0.78	0.68–0.89	**<0.0001**
miR-455-3p	−1.01	0.0281	**0.86**	0.78–0.94	**<0.0001**
miR-492	−2.92	0.0001	0.75	0.62–0.87	**<0.0001**
miR-566	−2.78	<0.0001	**0.84**	0.73–0.95	**<0.0001**
miR-624	1.81	0.0182	0.72	0.58–0.85	**0.0035**
miR-636	−1.75	<0.0001	0.74	0.62–0.85	**0.0014**
miR-920	1.69	0.0357	0.77	0.65–0.89	**0.00023**

*AUC, area under ROC curve; 95% CI, 95% confidence interval; FC, fold change. Bold values of 0.84, 0.83, 0.86, and 0.84 represent the AUC of miRNAs. Bold values indicates statistically significant difference P < 0.05*

### Promising Relative Expression Levels of miRNAs and Diagnostic Value Assessed in a Validation Cohort

**Figure 2:**
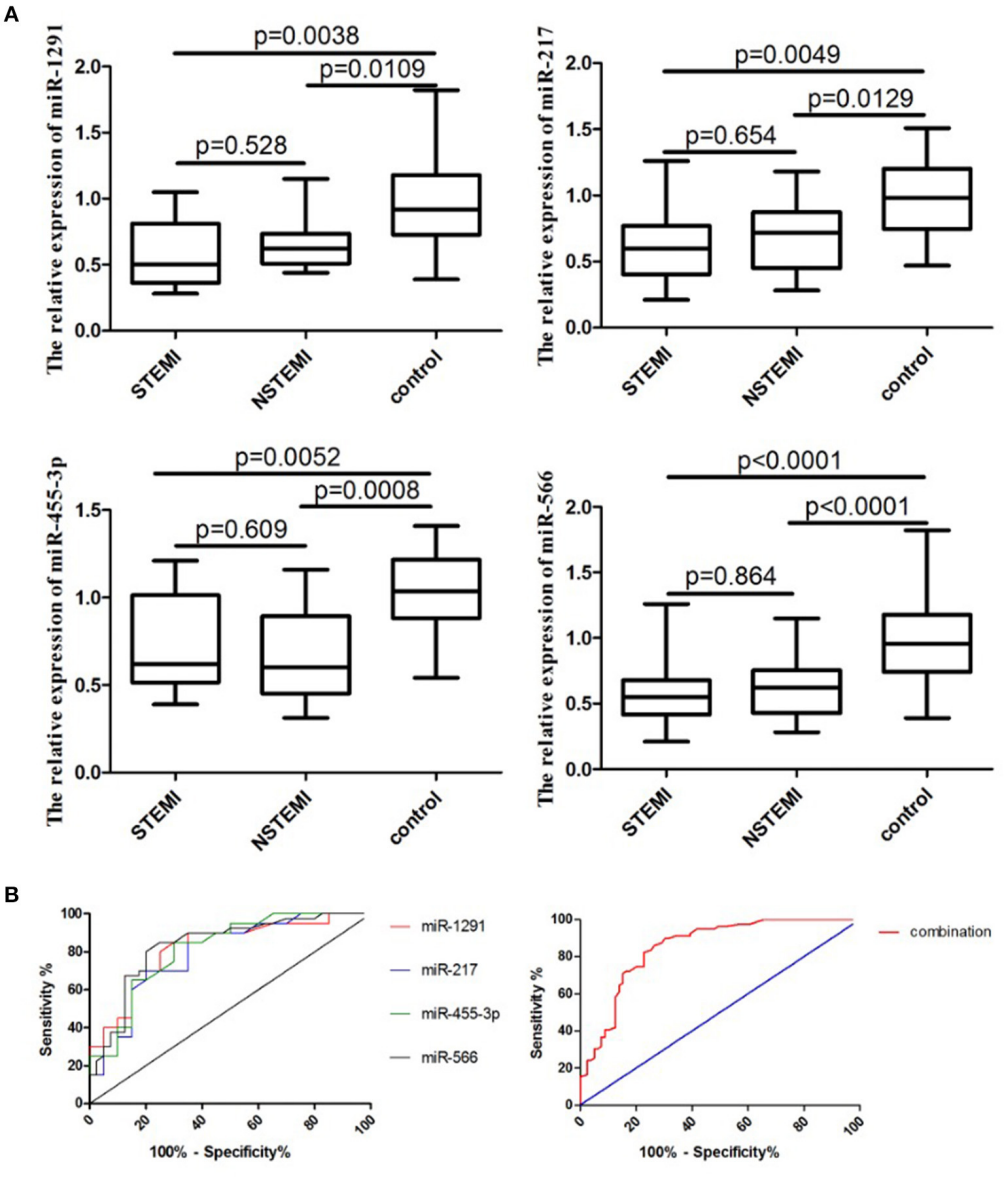
Expression of a four-miRNA panel in this cohort study. **(A)** Relative expression of miR-1291, miR-217, miR-455-3p, and miR-566 in the plasma of patients with AMI (ST-segment-elevation myocardial infarction, STEMI and non-ST-segment elevation myocardial infarction, NSTEMI). **(B)** ROC curves of miR-1291, miR-217, miR-455-3p, and miR-566, and the combination of four miRNAs in the validation phase.

A larger cohort comprising 80 patients with AMI and 80 controls was used to further validate the diagnostic role of miR-1291, miR-217, miR-455-3p, and miR-566. As shown in Figure 2A, the relative expression levels of miR-1291, miR-217, miR-455-3p, and miR-566 were significantly downregulated in the plasma of patients with AMI, when compared with controls. The ROC curve revealed that the AUC was 0.82 (95% CI: 0.69–0.95, *P* = 0.0005) for miR-1291, 0.79 (95%CI: 0.65–0.93, *P* = 0.0017) for miR-217, 0.82 (95% CI: 0.68–0.95, *P* = 0.0007) for miR-455-3p, 0.83 (95% CI: 0.74–0.93, *P* < 0.0001) for miR-566, and 0.87 (95% CI: 0.80–0.93, *P* < 0.0001) for the four-miRNA combination (Figure 2B). The sensitivity and specificity of the four-miRNA combination used for the diagnosis of AMI were 83 and 87%, respectively.

### Relative Expression Peaks of miR-1291, miR-217, miR-455-3p, miR-566, CK-MB, and cTnI Monitored After Onset of AMI

**Figure 3:**
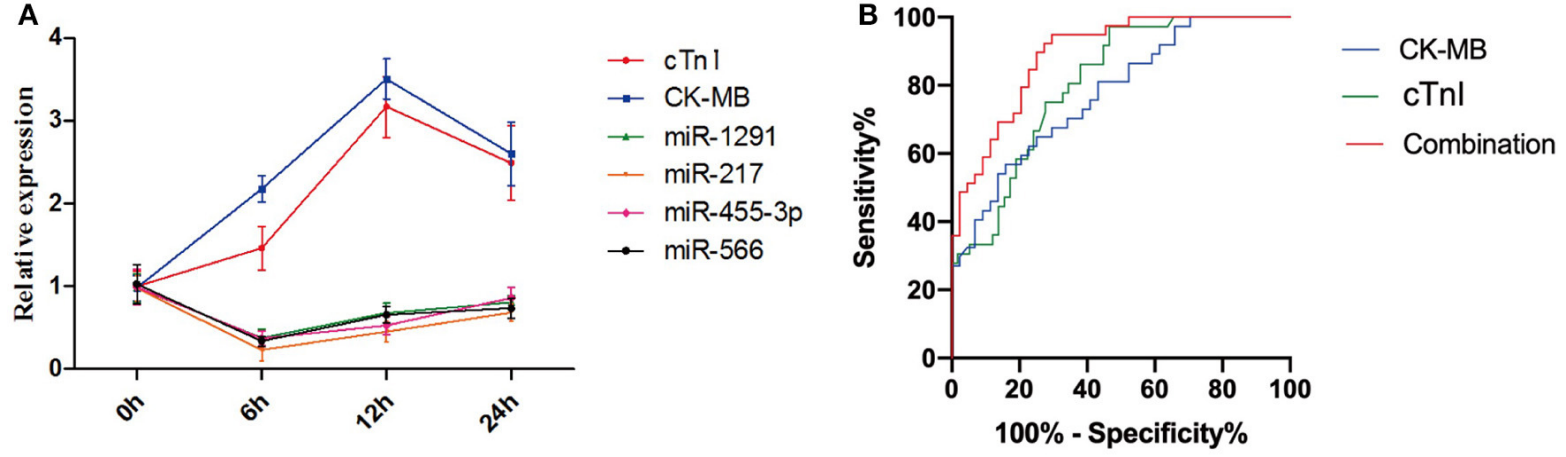
**(A)** Relative expression peaks of miR-1291, miR-217, miR-455-3p, miR-566, CK-MB, and cTnI after the onset of AMI. **(B)** ROC curves of CK-MB, cTnI, and the combination of four miRNAs, CK-MB, and cTnI.

As shown in Figure 3A, the expression levels of miR-1291, miR-217, miR-455-3p, miR-566, CK-MB, and cTnI in patients with AMI started to change since admission. However, the relative expression levels of miR-1291, miR-217, miR-455-3p, and miR-566 peaked 6 h following onset of AMI, which was earlier than the peaks of CK-MB and cTnI. Given that CK-MB and cTnI are still the key indicators to diagnose patients with AMI, we hypothesized that it may be better to combine them with the miRNAs set. As expected, the ROC curve revealed that the AUC value combining the four miRNAs set with CK-MB, and cTnI was 0.91 (95% CI: 0.84–0.98, *P* < 0.0001), which was higher than that of CK-MB (0.78, 95% CI: 0.68–0.88, *P* < 0.0001) and cTnI (0.81, 95% CI: 0.72–0.90, *P* < 0.0001) alone (Figure 3B). The sensitivity and specificity to diagnose AMI of combining the four miRNAs set with CK-MB and cTnI were 91 and 78%, respectively.

### Bioinformatics Analysis Revealed miRNAs Targets Associated With AMI

**Figure 4:**
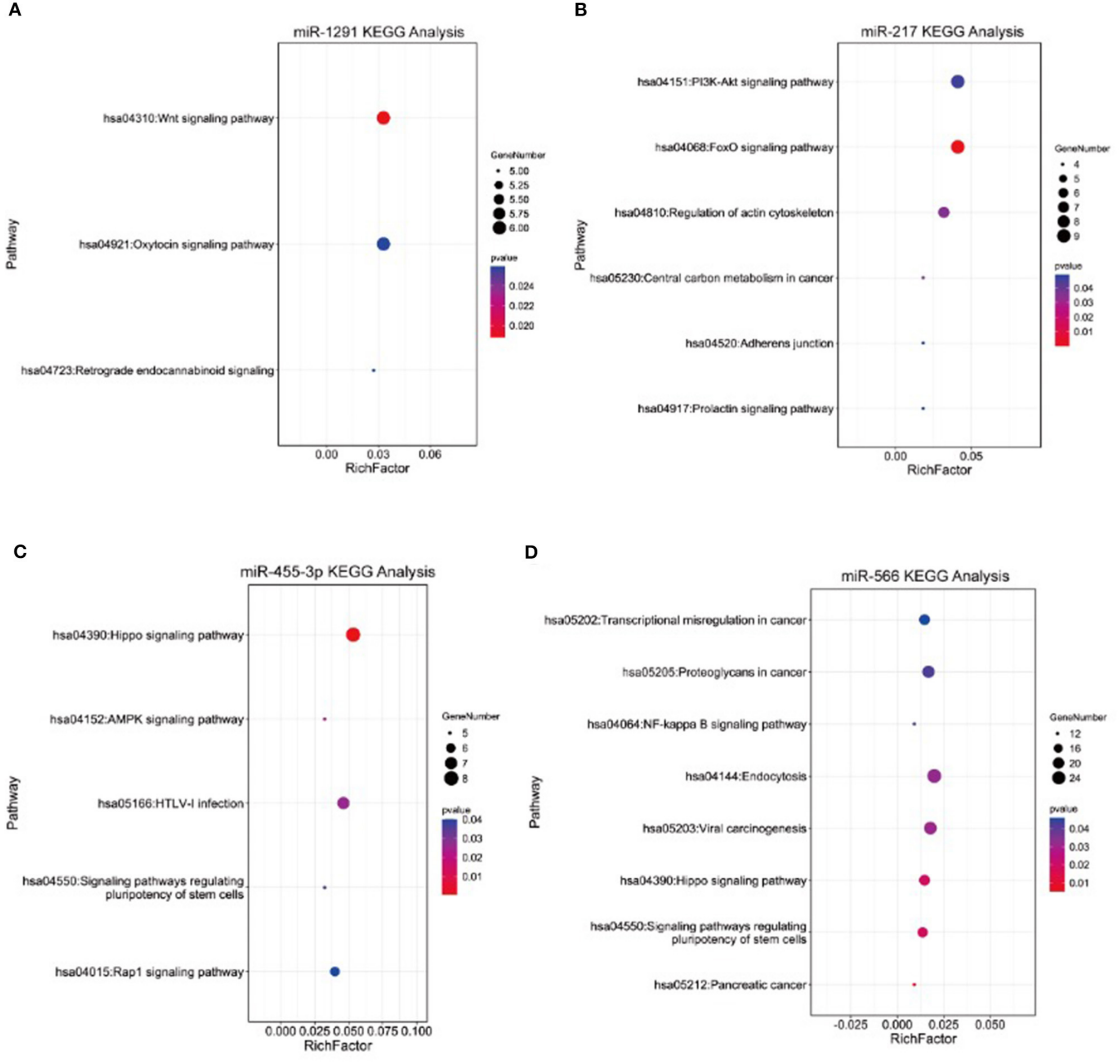
Kyoto Encyclopedia of Genes and Genomes (KEGG) pathway analysis of targets of **(A)** miR-1291, **(B)** miR-217, **(C)** miR-455-3p, and **(D)** miR-566.

To further explore the potential association between the four miRNAs and AMI, we conducted bioinformatic analyses. The targets of miRNAs were searched by integrated analysis of TargetScan 7.2 database and miRDB database. KEGG pathway analysis showed that the targets of four miRNAs were significantly enriched in several signaling pathways, which were associated with AMI progression, including the NF-kappa B signaling pathway, the Hippo signaling pathway, the Wnt signaling pathway, the PI3K-Akt signaling pathway, and the AMPK signaling pathway (Figure 4).

## Discussion

Acute myocardial infarction is one of the major causes of death worldwide. The early diagnosis of AMI can help patients access therapy more rapidly, thereby improving their survival rate (Yeh et al., 2010). AMI diagnosis is currently based on the expression of circulating cTn and CK-MB (Rapezzi et al., 2008). However, the use of such molecules as diagnostic biomarkers has some disadvantages, including not being timely enough. Therefore, novel biomarkers are urgently needed.

Recently, miRNAs have been reported to be significantly associated with cardiovascular diseases. They can modulate cardiac muscle cell growth, thus participating in the occurrence and progression of myocardial thickness, injury, remodeling, and heart failure (Wang et al., 2016b). Same MiRNAs can be derived from a variety of cell sources and can ultimately be secreted in the blood. Secreted miRNAs can be encapsulated in microparticles (exosomes, microvesicles, and apoptotic bodies), or combined with proteins or HDL (Navickas et al., 2016). Noticeably, Bayoumi et al. (2018) demonstrated that miR-125b-5p protected the heart from AMI by repressing pro-apoptotic genes BCL2 antagonist/killer 1 (bak1) and Kruppel like factor 13 (klf13) in cardiomyocytes. In addition, Li et al. (2018) discovered that miRNA-23a regulated AMI in patients and *in vitro* through targeting phosphatase and tensin homolog (PTEN). Also, Huang et al. (2016) declared that miR-103a targeted Piezo1 and was involved in AMI through regulating endothelium function. Evidently, miRNAs have a preponderant role during AMI.

Furthermore, the use of circulating miRNAs, including miR-486, miR-150, miR-19b-3p, miR-134-5p, miR-186-5p, miR-21, miR-208a, miR-124, and miR-221-3p, as diagnostic markers has been widely investigated (Bialek et al., 2015; Zhang et al., 2015; Coskunpinar et al., 2016; Wang et al., 2016a; Guo et al., 2017). Importantly, Zeller et al. (2014) demonstrated that the diagnostic accuracy of miR-132, miR-150, and miR-186 was improved when applied as a miRNAs panel. At the same time, Usuba et al. (2019) found that together a group of circulating miRNAs was more specific in the early detection of bladder cancer. These studies showed the superiority of a panel of miRNAs against single ones.

In this study, a panel of four miRNAs (miR-1291, miR-217, miR-455-3p, and miR-566) was identified as a novel diagnostic biomarker for AMI after an integrated analysis of two microarray results and a comparison of their AUC values using ROC analysis. Regarding the four miRNAs set, miR-1291 was found to have regulatory effects in many tumors (Escuin et al., 2020). On its side, miR-217 inhibits apoptosis of atherosclerotic endothelial cells *via* the TLR4/PI3K/Akt/NF-κB pathway (Jiang et al., 2020). Interestingly, Li et al. (2016) also found that atherosclerotic-related miRNAs led to an early diagnosis of AMI. Relatively to miR-455-3p, it is reported to reduce apoptosis and alleviate degeneration of chondrocytes through regulating the PI3K/AKT pathway (Wen et al., 2020). Meanwhile, miR-566 mediates cell migration and invasion in colon cancer cells by direct targeting of PSKH1 (Pan et al., 2018). Peripheral blood concentrations of miR-1291, miR-217, miR-455-3p, miR-566, CK-MB, and cTnI were measured at different time points following the initiation of chest pain to determine the moment when they reach their highest peak. The results showed that circulating miR-1291, miR-217, miR-455-3p, and miR-566 levels peaked 6 h after the onset of chest pain; whereas, cTnI and CK-MB levels peaked after 12 h. It also highlighted the potential of the miRNA panel as an early diagnostic biomarker of AMI. Together with its high AUC, these miRNAs panel could provide patients with AMI with early diagnostic information after admission, thus distinguishing, as soon as possible, AMI from other diseases involving chest pain. It is well-known that CK-MB and cTnI are released when myocardium necrosis occurs (Bentzon et al., 2014). At this time, myocardial infarction is relatively late and irreversible, while plaque rupture and platelet activation are early events of thrombosis that induce AMI (Li et al., 2019). Hence, miRNAs associated with these processes may be released earlier than CK-MB and cTnI; furthermore, Oerlemans et al. (2012) revealed that miRNAs release faster due to not only passive release but also active secretion. Therefore, circulating miRNAs can be secreted at an early stage of the disease. In this study, we hypothesized that the potential of thesefour miRNAs in the early detection of AMI may be associated with plaque rupture and platelet activation. Indeed, the four miRNAs might be enriched in plaques and platelets. Given that CK-MB and cTnI are still the key indicators to diagnose patients with AMI, we also hypothesized that it may be better to combine them with the miRNAs set. Early diagnosis and proper treatment of AMI could strongly reduce mortality and improve the survival rate of patients (White and Chew, 2008).

Finally, to explore the potential association between the four miRNAs and AMI, we conducted bioinformatic analyses. KEGG pathway analysis showed that the four miRNAs were significantly enriched in several signaling pathways, which were associated with AMI progression, including the NF-kappa B signaling pathway, the Hippo signaling pathway, the Wnt signaling pathway, the PI3K-Akt signaling pathway, and the AMPK signaling pathway. Ge et al. (2019) reported that silencing the IRAK3 gene can prevent cardiac rupture and ventricular remodeling through the NF-κB signaling pathway in a mouse model of AMI. Liu (2020) discovered that LncRNA-P21 regulates the progression of AMI *via* the Wnt/β-catenin signaling pathway. In addition, the PI3K/Akt signaling pathway is also an important pathway to modify the myocardial apoptosis and cardiac function in rats with AMI (Chen et al., 2021). In addition to their biomarker role of AMI, the regulatory mechanisms of miR-1291, miR-217, miR-455-3p, and miR-566 in AMI progression deserve to be further investigated.

Overall, this study had several limitations: (1) this was a single-center investigation, and the conclusions might not be easily extrapolated to other locations; (2) the sample size was relatively small, and, hence, a larger cohort with more specimens was needed; and (3) the selection of identified miRNAs came from the analysis of GEO dataset, thus ignoring other miRNAs that could hold potential as AMI diagnostic biomarker too.

However, this work shows the superiority of a group of miRNAs against single ones. They can be used as a “signature” of some pathological conditions, such as AMI. A panel of four miRNAs, namely miR-1291, miR-217, miR-455-3p, and miR-566, were identified as a promising early diagnostic tool for AMI condition, either with STEMI or NSTEMI electrocardiograms. Their expression appeared to be downregulated in peripheral blood samples during AMI. This article highlights the importance of circulating miRNAs during cardiovascular diseases. Further research to understand their origin, role, and potential as a biomarker or therapeutic targets should continue forward. To summarize, we identified a four-miRNA panel in peripheral blood that is downregulated during AMI and can act as a potential early diagnostic biomarker.

## Data Availability Statement

The raw data supporting the conclusions of this article will be made available by the authors, without undue reservation.

## Ethics Statement

The studies involving human participants were reviewed and approved by the ethical committee of Second Affiliated Hospital of Nantong University (2019KN109). The patients/participants provided their written informed consent to participate in this study.

## Author Contributions

LC and HL designed this study and wrote this paper. KZ designed this study conducted qRT-PCR of miRNAs and analyze GEO database. JL conducted qRT-PCR of miRNAs and collected the biochemical results of serum of participants. JB conducted qRT-PCR of miRNAs. All authors contributed to the article and approved the submitted version.

## Conflict of Interest

The authors declare that the research was conducted in the absence of any commercial or financial relationships that could be construed as a potential conflict of interest.

## Abbreviations

AMI: acute myocardial infarction
CK-MB: creatine kinase-MB
cTnI: cardiac troponin I
DBP: diastolic blood pressure
HDL: high-density lipoprotein
LDL: low-density lipoprotein
NSTEMI: non-ST-segment elevation myocardial infarction
SBP: systolic blood pressure
STEMI: ST-segment-elevation myocardial infarction
*P*-value: comparison between patients with AMI and healthy controls.
